# Genomics of post-bottleneck recovery in the northern elephant seal

**DOI:** 10.1038/s41559-024-02337-4

**Published:** 2024-02-21

**Authors:** A. Rus Hoelzel, Georgios A. Gkafas, Hui Kang, Fatih Sarigol, Burney Le Boeuf, Daniel P. Costa, Roxanne S. Beltran, Joanne Reiter, Patrick W. Robinson, Nancy McInerney, Inge Seim, Shuai Sun, Guangyi Fan, Songhai Li

**Affiliations:** 1 Biosciences, Durham University, Durham, UK; 2 Department of Ichthyology and Aquatic Environment, University of Thessaly, Volos, Greece; 3 Marine Mammal and Marine Bioacoustics Laboratory, Institute of Deep-sea Science and Engineering, Chinese Academy of Sciences, Sanya, China; 4 Innovation Research Center for Aquatic Mammals, Institute of Hydrobiology, Chinese Academy of Sciences, Wuhan, China; 5 Ecology and Evolutionary Biology, University of California, Santa Cruz, CA USA; 6 Center for Conservation Genomics, National Zoo and Conservation Biology Institute, Smithsonian Institution, Washington, DC USA; 7 Integrative Biology Laboratory, College of Life Sciences, Nanjing Normal University, Nanjing, China; 8 BGI Research, Qingdao, China

**Keywords:** Conservation biology, Population genetics, Molecular evolution

## Abstract

Populations and species are threatened by human pressure, but their fate is variable. Some depleted populations, such as that of the northern elephant seal (*Mirounga angustirostris*), recover rapidly even when the surviving population was small. The northern elephant seal was hunted extensively and taken by collectors between the early 1800s and 1892, suffering an extreme population bottleneck as a consequence. Recovery was rapid and now there are over 200,000 individuals. We sequenced 260 modern and 8 historical northern elephant seal nuclear genomes to assess the impact of the population bottleneck on individual northern elephant seals and to better understand their recovery. Here we show that inbreeding, an increase in the frequency of alleles compromised by lost function, and allele frequency distortion, reduced the fitness of breeding males and females, as well as the performance of adult females on foraging migrations. We provide a detailed investigation of the impact of a severe bottleneck on fitness at the genomic level and report on the role of specific gene systems.

## Abstract (web summary)

Genome analysis of modern and historical elephant seals reveals impacts of a severe bottleneck on the genomes and fitness of individual seals, and the implications for recovery.

## Main

Iconic species are vanishing. Our past and present activities have reduced populations to the point where they may go extinct by demographic processes alone^1^. When they survive, inbreeding and genetic drift may reduce the fitness of individuals and the survival potential of populations^2^. Nevertheless, some species survive and apparently thrive. After heavy exploitation led to a severe population bottleneck in 1892, reducing the population to ~20 individuals^3^, the northern elephant seal (*Mirounga angustirostris*; hereafter NES) recovered nearly exponentially to over 220,000 today^4,5^. Theoretically, a rapid demographic recovery may reduce the negative impact on genetic diversity, but this was not the case for the NES. Census data indicate a rapid recovery^4,5^, but years of genetic studies show profoundly reduced genetic diversity^6^. In one of the earliest indications of the impact of population bottlenecks on the health of a species, ref. ^7^ reported the lack of protein (allozyme) diversity in the surviving population of NES. Further studies on genetic diversity at a range of markers (allozymes, mitochondiral (mt)DNA, minisatellite DNA, microsatellite DNA and immune system genes) showed reduced diversity compared with southern elephant seals (*Mirounga leonina*, a sibling species not impacted by a similar population bottleneck^3,6,8^). The loss of diversity was also evident in comparisons of pre- and post-bottleneck NES DNA^9,10^. Small population size compounded by extreme polygyny^8^ probably contributed to the loss of genetic diversity. Of 150,388 species reviewed for the IUCN Red List, 42,108 are threatened and 25,615 are endangered or critically endangered (http://www.iucnredlist.org). Many of the endangered or threatened species have previously existed or currently exist as small populations. Since the survival potential of small populations can be influenced by genetic diversity^2^, we wonder whether the loss of genetic diversity for NES has had a measurable effect on their fitness, even though population growth has been robust during the 132 years (~22 generations) since the bottleneck. If so, they could still be vulnerable to some new environmental stress.

Here we sequenced 260 modern and 8 historical genomes, showing that inbreeding, loss of function and the distortion of allele frequencies have reduced the fitness of breeding males and females, as well as the performance of adults on foraging migrations. The loss of fitness associated with inbreeding is well documented^11^, but the importance of allele frequency distortion and the presence of loss of function (LOF) alleles in specific gene systems is less well understood and provides new inference about the general and lasting impact of population bottlenecks. Ecosystem function depends on biodiversity and the contribution of species within that system. Effective conservation management requires an understanding of the scope of impact from depleted populations on specific functions in ecosystem communities.

## Results and Discussion

All of the modern NES samples investigated in our study were from the breeding colony at Año Nuevo, California, USA. They were chosen due to their inclusion in studies on reproductive success or diving profiles. The source of historical samples is given in Supplementary Table 1. We consider diversity across NES genomes, comparing them before and after the bottleneck, followed by inference from the genomic analyses about demographic history. We then consider fitness, first associated with reproductive success, then with diving performance.

### Diversity

**Fig. 1:**
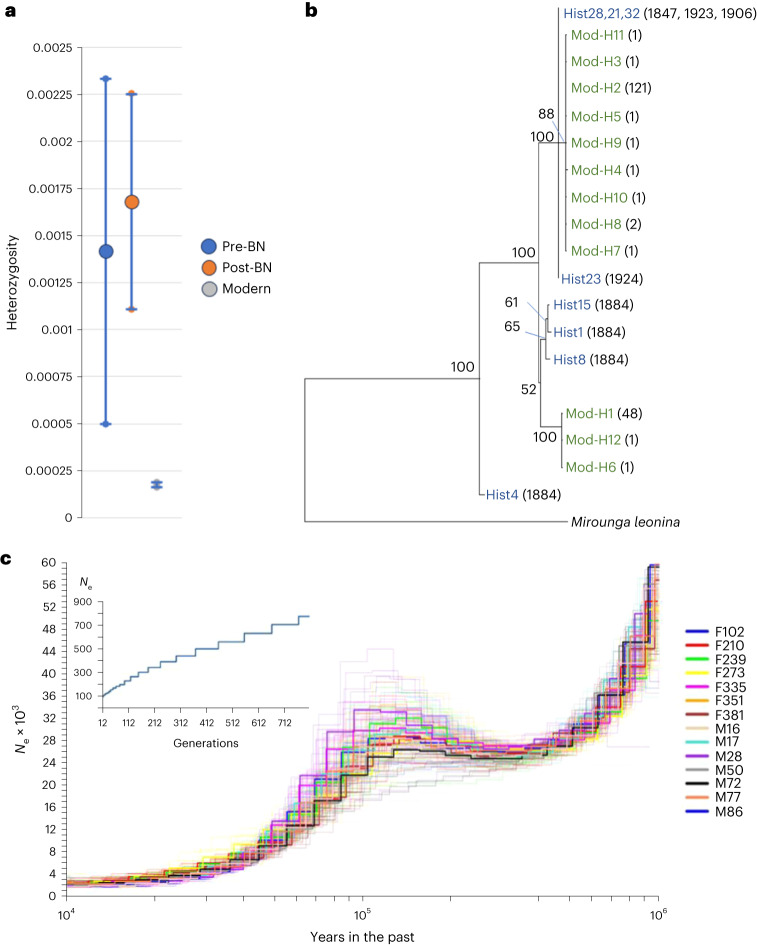
Diversity and demographics. **a**, Mean (±1 s.d.) nuclear heterozygosity for pre (*N* = 5), immediately post (1906–1923; *N* = 3) bottleneck (BN) and modern genomes (*N* = 180). **b**, Mitogenome DNA neighbour-joining tree comparing historical (blue) and modern (green) haplotypes for all historical and all modern adult samples. For the historical samples (Hist), numbers in brackets indicate dates; for the modern samples, numbers in brackets indicate the numbers of individuals with a given haplotype. Bootstrap values reflect proportional support (%) out of 1,000 replicates. **c**, PSMC plot for 14 modern samples showing historical demography (pale lines showing bootstrap replicates). Inset: effective population size (*N*_e_) estimate based on linkage disequilibrium. Source data

**Extended Data Fig. 1:**
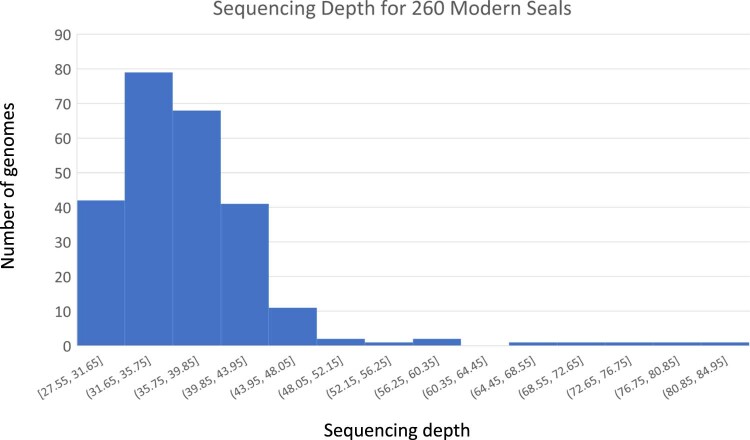
Histogram showing the distribution of read depths for the 260 modern samples. Source data

**Extended Data Fig. 2:**
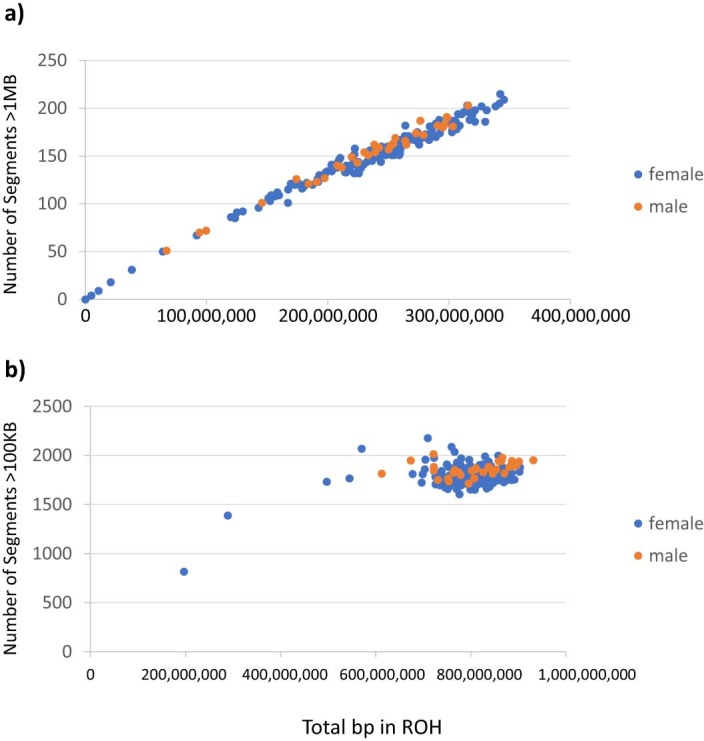
ROH number of segments compared to total bp covered. a) fragments > 1MB, and b) fragments greater than 100KB. Source data

**Extended Data Fig. 3:**
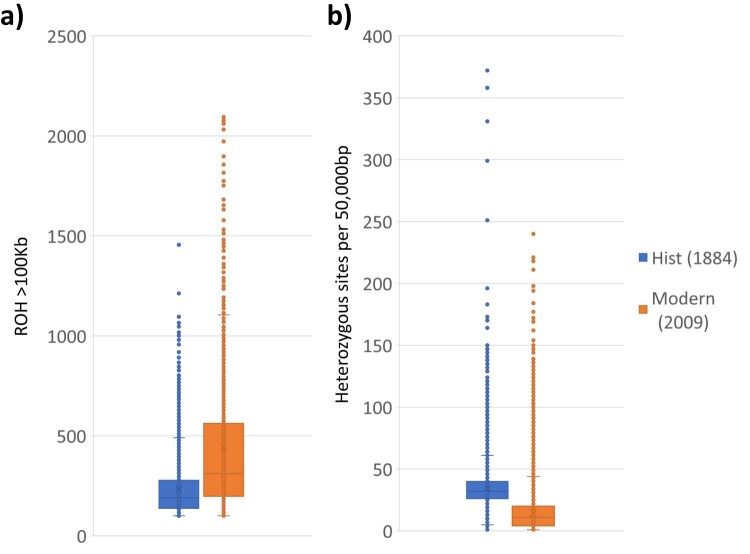
Comparison of a historical (Hist8) and a modern (sample 1234) nuclear genomes. a) The number of segments of ROH of different sizes greater than 100Kb. b) The number of heterozygous sites in 50,000 bp sliding windows. For each illustration, the box represented the inter-quartile range (50% of the data), the horizotal line through the box is the median, the whiskers include 75% of the data, and the remaining dots are outside that range. Source data

Most of our modern nuclear genome sequences had greater than 30X read depth (see Extended Data Fig. 1 for full range), while 8 historical genomes with degraded DNA had a broader range of coverage (14.5X ± 12.7X after mapping; Supplementary Table 1). Average heterozygosity per genome was 0.00142 ± 0.00092 (s.d.) before the bottleneck (*N* = 5) and 0.000176 ± 0.000013 in the modern population (*N* = 180 adults; Fig. 1). The size range and number of runs of homozygosity (ROH) fragments >100 kb and >1 Mb are shown for male and female modern samples in Extended Data Fig. 2. Coverage was limited for most of the historical samples, restricting the potential for accurately estimating ROH. However, a pairwise comparison for both heterozygosity per sliding 50 kb window and ROH greater than 100 kb is shown in Extended Data Fig. 3, comparing the genomes of two individuals, one from 1884 (Hist8) that sequenced well enough for these analyses and a randomly chosen modern sample from 2009. The individual from 1884 was an adult male. His diversity reflects the population of his parents, who were alive when the species was sufficiently abundant to support a commercial hunt. Each comparison shows a loss of variation after the bottleneck. For this historical genome, the proportion of genotypes homozygous for the LOF allele (among all LOF loci identified by snpEff, see Methods) was 0.0011, while the proportion of heterozygotes was 0.287. Among 180 modern adult genomes, 0.379 ± 0.016 (s.d.) of the identified loci were homozygous for the LOF allele, while 0.193 ± 0.024 were heterozygous.

A mitogenome tree was constructed with the 5 pre-bottleneck samples, compared to 3 from shortly after the bottleneck (1906–1924) and 180 modern genomes (Fig. 1). Two major lineages remained after the bottleneck, and some mutations were gained in the modern population within those two lineages. The pre-bottleneck mitochondrial genomes had an average pairwise genetic distance (uncorrected percentage) between them of 0.00219 ± 0.00146 (s.d.). The two main modern haplotypes remaining (haplotypes 1 and 2; Fig. 1) differ by 0.00232, while diversity within each lineage was 0.0000048 in the haplotype 1 lineage and 0.0000081 in the haplotype 2 lineage. The tree indicates that two mtDNA lineages survived the bottleneck, consistent with earlier reports^3^.

### Demographic history

**Extended Data Fig. 4:**
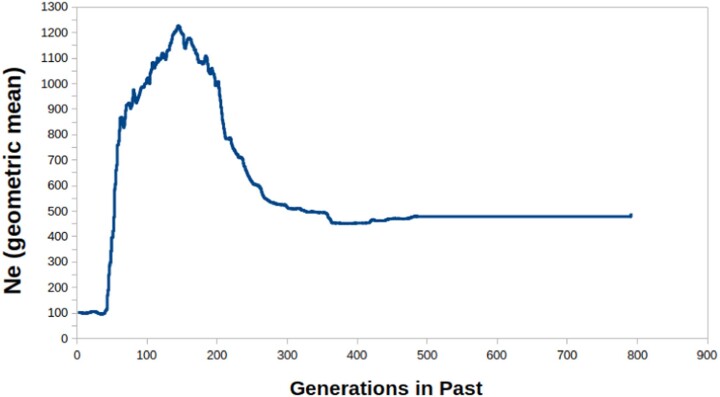
Historical demographic profile estimated using the program GONE. Source data

The demographic history of the species can be estimated in deep time on the basis of coalescent analyses using genomes (with the pairwise sequential Markovian coalescent (PSMC), see Methods). The 14 modern genomes we chose at random (while ensuring 7 males and 7 females, all from the 1980s) all showed essentially the same pattern (Fig. 1). The effective population size (*N*_e_) was ~40,000 during the last interglacial (Eemian; ~130–115 Ka) but fell to ~2,000–4,000 during the last glacial period, reaching a nadir around the last glacial maximum (~20 Ka; Fig. 1). Using the approximate effective to census population size (*N*_e_/*N*_c_)ratio published from a meta-analysis^12^ of wildlife species (~0.1), this would suggest a census population of ~20,000, perhaps explaining why the population was so quickly nearly eradicated during the nineteenth century. The population may have been closer to its current size during the Eemian. For an estimate of current *N*_e_ (post-bottleneck), we used a method^13^ based on linkage disequilibrium (SNeP; Fig. 1c), which indicated an *N*_e_ of 100, suggesting a very low *N*_e_/*N*_c_ ratio (~0.00045), consistent with low post-bottleneck diversity and rapid regrowth. This result was replicated with an alternative method (GONE)^14^, which indicated the same current *N*_e_ (see Extended Data Fig. 4).

### Reproductive fitness

**Fig. 2:**
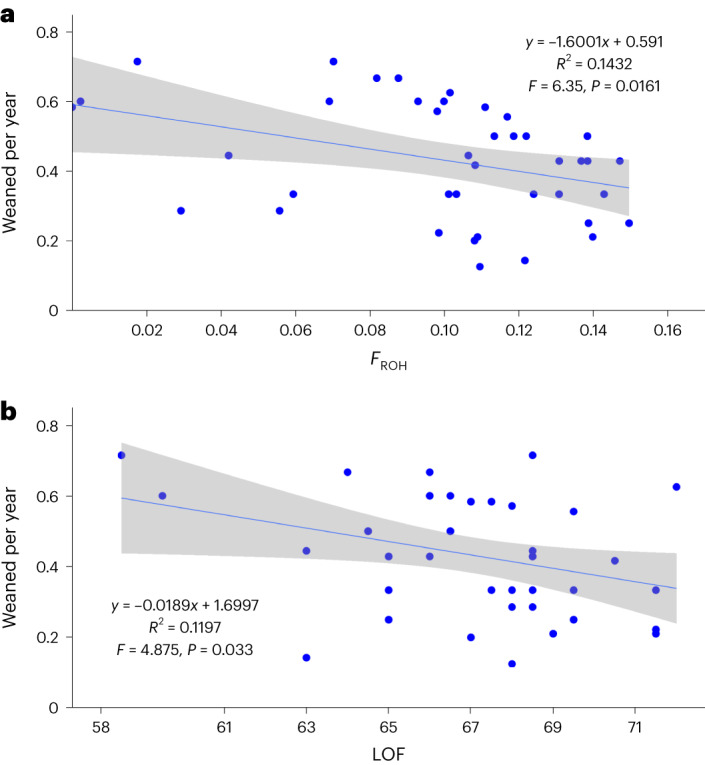
Reproductive fitness, inbreeding and LOF. Pups weaned annually over the female’s lifetime as a measure of lifetime success is compared by linear regression with **a**, proportional runs of homozygosity (*F*_ROH_) and **b**, the number of affected alleles across 200 LOF loci. Regression details and significance are shown for each. The 95% confidence intervals (shown in grey shading) were calculated using the R package ggplot. Source data

**Extended Data Fig. 5:**
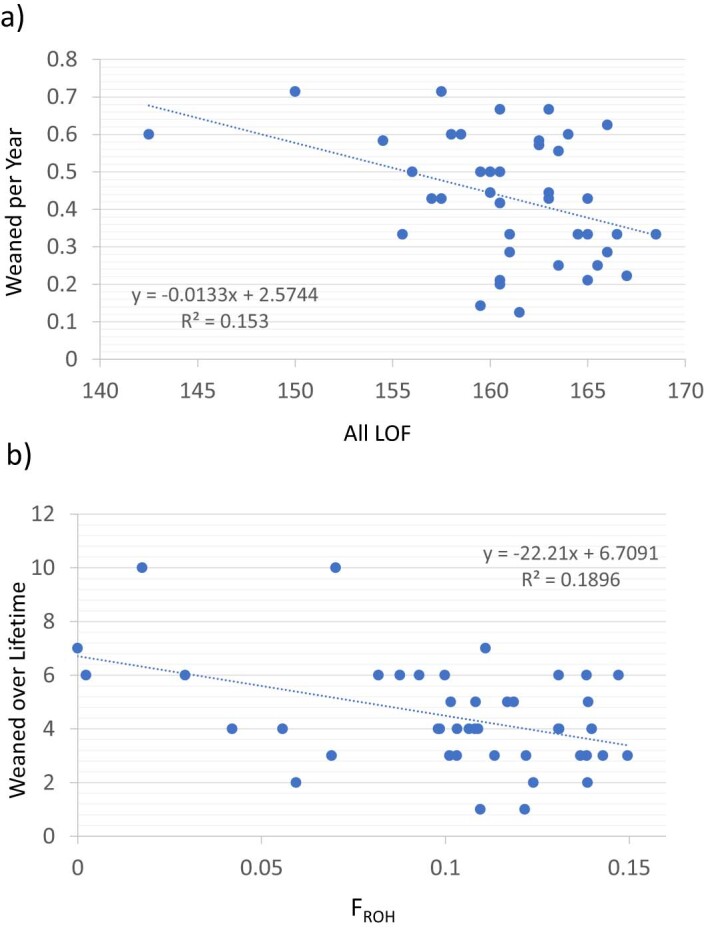
Fitness correlations with total LOF and all weaned. a) Correlation between all LOF loci reported from snpEff (weak and strong effect) against weaner success per year. b) Correlation between FROH and the total pups weaned over a lifetime. Source data

The average number of successfully weaned pups per year over a female’s lifetime was known for 40 females in our dataset. This fitness estimate reflects the lifetime contribution to future generations, and numerous studies have considered correlations between heterozygosity and fitness to assess the impact of inbreeding or heterosis (see ref. ^15^). We tested for correlation with *F*_ROH_ (for ROH > 1Mb; *r*^2^ = 0.143, *F* = 6.35, *P* = 0.016) and with the number of affected alleles across 151 LOF loci (restricted to loss of start or new stop mutations; *r*^2^ = 0.114, *F* = 4.88, *P* = 0.033; Fig. 2). The regression was also significant for all 328 LOF loci found (*r*^2^ = 0.146, *F* = 6.49, *P* = 0.015; Extended Data Fig. 5). The correlation with *F*_ROH_ was marginally stronger when the total number of weaned pups over a lifetime was considered (*N* = 43; *r*^2^ = 0.19, *F* = 9.59, *P* = 0.0035; Extended Data Fig. 5), which does not control for variation in longevity, but longevity itself was not significantly correlated (*N* = 44; *r*^2^ = 0.024; *F* = 1.01; *P* = 0.32). There was also no significant correlation between *F*_ROH_ and the number of pups a female produced (fecundity; *N* = 44, *r*^2^ = 0.07, *F* = 3.16, *P* = 0.083). We considered possible environmental effects, although females are all from a very similar time range, born between 1981 and 1988, and first weaning between 1985 and 1991. A multiple regression with lifetime reproductive success (weaned per year) as the response variable, and female birth year and year of first weaning as explanatory variables, was not significant (adjusted *r*^2^ = −0.0096, *F* = 0.81, *P* = 0.45). When we included *F*_ROH_ as a third explanatory variable, *F*_ROH_ was significantly correlated with the response variable (*P* = 0.03), but none of the interaction factors between explanatory variables were significant (Supplementary Table 2).

**Extended Data Fig. 6:**
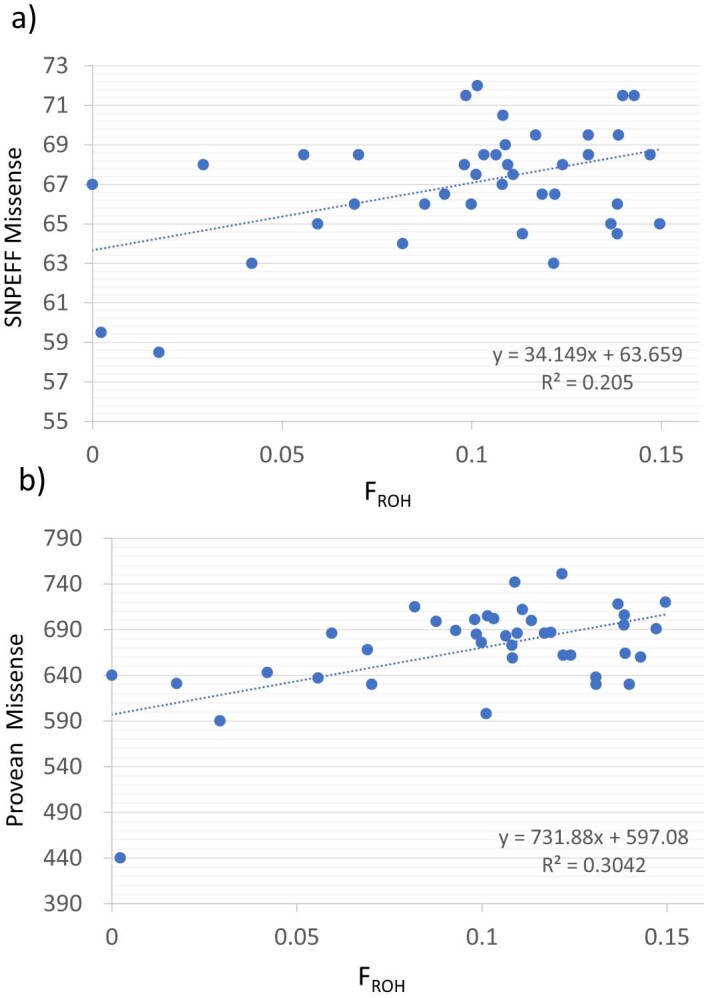
Correlations between LOF and FROH. Linear regression correlations between FROH ( > 1 MB) and a) loss of function based on the analysis in snpEff, and b) missense mutations estimated in PROVEAN. Source data

A significant correlation for pups weaned, but not for pups produced, suggests that the impairment may be more about the fitness of offspring affected by inbreeding^16^ rather than fecundity; however, more data on the genotypes and phenotypes of the relevant pups would be needed to test this further. The linear regression between the number of LOF loci and *F*_ROH_ (*x* axis) was positive and significant (*F* = 9.78, *P* = 0.0034, *r*^2^ = 0.3042; Extended Data Fig. 6), as expected. We also used an additional programme (PROVEAN; https://www.jcvi.org/research/provean) to identify missense mutations and confirm a positive correlation with *F*_ROH_ (*F* = 16.61, *P* = 0.0002; Extended Data Fig. 6). Three LOF loci were associated with oogenesis (MARF1)^17^, oocyte growth (KMT2B)^18^ and embryonic development (NBAS)^19^, but the profile among these loci was the same for all but three females, and there was no association with fitness. An association with diversity across the genome rather than specific loci would be consistent with the ‘general effect’ hypothesis of heterozygosity–fitness correlations^20^.

**Extended Data Fig. 7:**
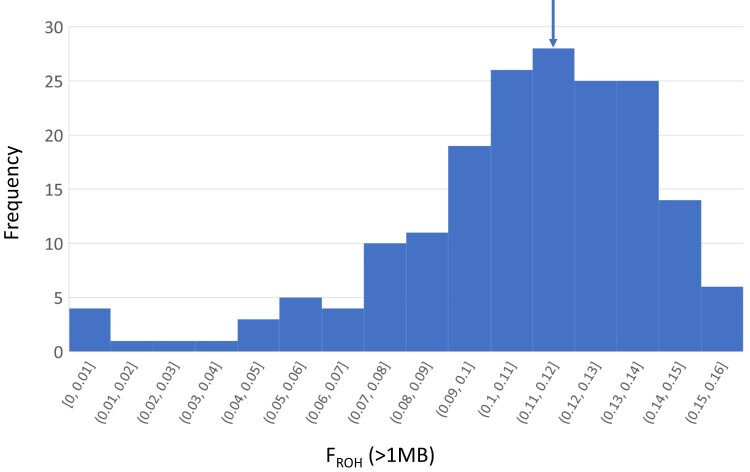
Relative proportion of runs of homozygosity greater than 1 MB in different range categories (in brackets) for all adult individuals, showing level found for male M12 (arrow). Source data

**Extended Data Fig. 8:**
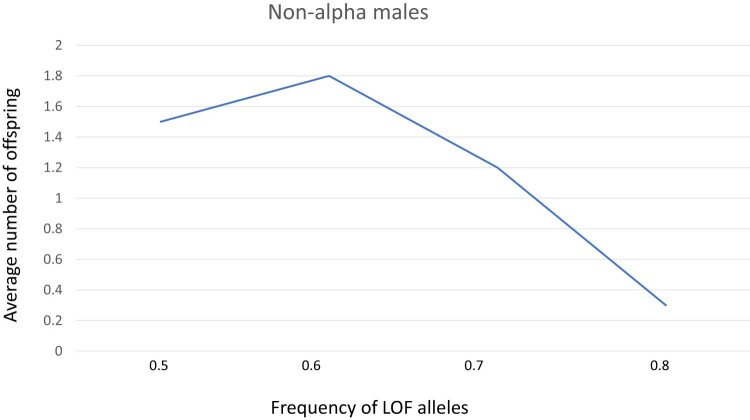
Relationship between the number of paternities achieved and the frequencuy of loss of function alleles at loci associated with male health in non-alpha males. Source data

We next consider a possible impact on male reproductive success. In an earlier study, the paternal success of the NES alpha male M12 was low compared with that expected by his frequency of observed copulations^21^. That study had investigated 10 NES harems, all from the same beach and same year, and 6 southern elephant seal (SES) harems. The average paternal success of alpha bulls was significantly lower than copulatory success for NES but not for SES. One NES male stood out as having especially low success (M12). Here we used genomic data to test the paternal success of alpha males at 4 of the same harems, including the harem held by M12. A total of 31 males and 77 pups were included in the paternity tests (including the 4 focal alphas) and there were 51 paternities detected. We found again that only M12 had significantly fewer paternities than expected based on observed copulations (Table 1). M12 also had the highest *F*_ROH_, although all 4 were near the middle of the distribution for all adult NES (see Extended Data Fig. 7). We found a total of 5 loci with LOF (gained a stop codon), known to be associated with male fertility (associated with sperm production or function). These were *LRGUK*^22^, *MNS1* (ref. ^23^), *TUBB4B*^24^, *SRSF3* (ref. ^25^) and *EZR*^26^. We focused first on homozygotes for the LOF allele in case there is some function from co-dominance. M12 was homozygous for the non-functional version of 4 of these 5 loci, while the other alphas were homozygous for the non-functional version at 1 or 2 loci (Table 1). The negative relationship between paternal success and the frequency of the affected alleles was also seen for 27 non-alpha males (Extended Data Fig. 8 and Table 1). Males gain access to females in this highly polygynous species through competition among males, hence age and overall size are important factors associated with successes. For this reason, a general relationship with inbreeding or loss of function across the genome may not be expected. We tested this by looking for a correlation between non-alpha male paternal success and both *F*_ROH_ and relative LOF allele frequency (out of 151 LOF loci found that generated a new start or a stop codon). Neither were significant (*F*_ROH_: *r*^2^ = 0.031, *F* = 0.79, *P* = 0.38; LOF: *r*^2^ = 0.057, *F* = 1.5, *P* = 0.23).

**Table 1:** Alpha male reproductive success

Harem^a^	BBS (M144)	TS (M160)	MBB (M12)	BMS (M4)
**% Copulations**	31.5	25.9	90.5	42.7
**% Paternity**	26.7	7.7	23.5	26.9
***N*** **(genomes)**	15	13	17	26
***χ*2**	0.13	2.0	**17.6**	1.36
***P***	0.72	0.16	**0.00003**	0.24
***F***_**ROH**_ **(**>**1Mb)**	0.1067	0.1093	**0.1145**	0.0967
**Homozygosity at LOF loci**	1/5	2/5	**4/5**	1/5
**LOF allele frequency**	0.6	0.6	**0.8**	0.6

^a^Four focal northern elephant seal harems (column headings) with the alpha male shown in brackets. The percentage of observed copulations from earlier studies is compared with calculated percent paternities determined by genomic analysis. The significance of the difference was tested using two-sided Chi-square test. The proportional *F*_ROH_ (indicating inbreeding) and loss of function at five identified loci that are associated with male reproductive health are given. Bold text highlights the results for M12.

These data suggest that males, including M12 and some of the 27 non-alpha males, were impacted by specific LOF loci, reducing their fertility. Deleterious alleles can be retained after allele frequencies are distorted and purifying selection is weakened by strong genetic drift. The most successful of the non-alpha males (two males with five paternities each; Extended Data Table 1) were homozygous at two or three LOF loci. Both of these males and all alphas apart from M12 had functional alleles at *EZR*. M12 was homozygous for the LOF allele. This locus is dysfunctional in male humans with asthenozoospermia (reduced sperm motility)^26^. We compared the number homozygotes for the LOF allele at these five loci (average = 2.14) for all non-alpha males that had at least one offspring with those that had no offspring (average = 2.77). The difference was marginally significant (Mann–Whitney *U* = −1.72, *P* = 0.0427).

### Diving performance

**Fig. 3:**
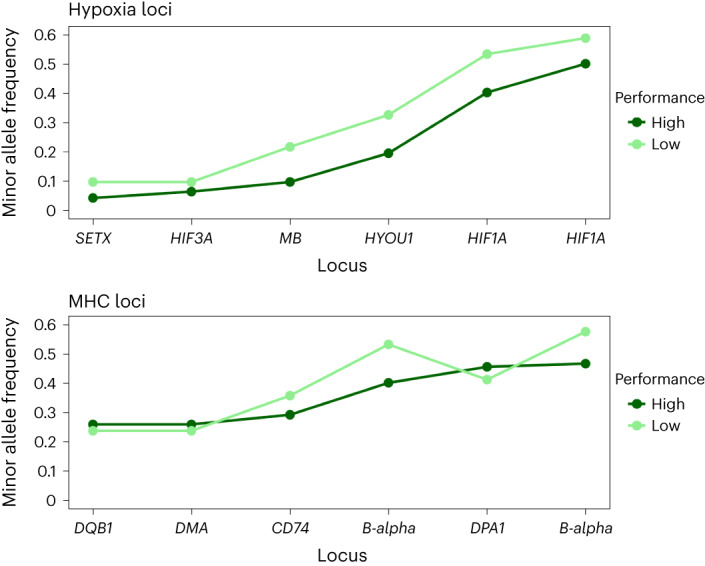
Fitness impact associated with deep diving. Minor allele frequencies at six SNP sites across five loci (*x* axis) associated with hypoxia (top) (see Table 2 for genotype frequencies) are compared with minor allele frequencies across six SNPs in five MHC loci (bottom) (Supplementary Table 3) for high and low dive performance. Source data

**Extended Data Fig. 9:**
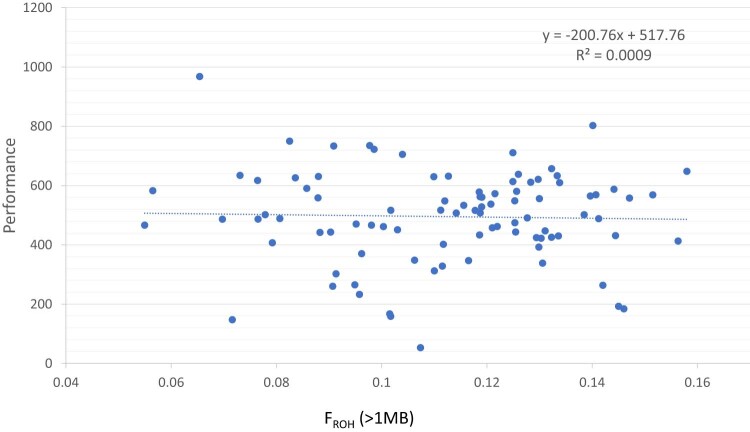
Relationship between the proportion of runs of homozygosity greater than 1 MB and the diving performance metric (no significant correlation). Source data

A critical aspect of elephant seal life history is their extensive deep-diving foraging excursions when they accumulate fat stores to facilitate fasting during the breeding season^27^. We acquired dive performance data from 92 females with complete dive profile data for up to three foraging trips per female lasting 70–220 d per trip. We generated a ‘performance’ metric as the product of the deepest dive (in metres, averaged over multiple trips) times the proportion of dives deeper than 516 m (median dive depth) times the relative dive duration (relative to the longest duration among individuals). Diving performance was not associated with metrics of genomic diversity (see Extended Data Fig. 9 for a correlation test with *F*_ROH_). We then categorized each seal as having high or low performance (either side of the midpoint). These categories were not differentiated with respect to LOF allele frequency (*t* = 1.20, d.f. = 90, *P* = 0.232 for the LOF loci resulting in lost start or new stop codons and *t* = 0.126, *P* = 0.90 for all LOF loci). We then compared these two categories at the five variable loci with either non-synonymous mutations or upstream mutations (that may be associated with transcriptional regulation) associated with hypoxia found in our genomes and supported by publications about their relevant function. Two hypoxia loci were variable at non-synonymous sites within the gene: *HIF3A* (involved in hypoxia gene expression)^28^ and *SETX* (protects cells from DNA damage induced during transcription in hypoxia)^29^. Three other loci had segregating sites upstream of the genes: *MB* (myoglobin; oxygen provision)^30^, *HIF1A* (transcription regulator in response to hypoxia)^30^ and *HYOU1* (cyto-protection during oxygen deprivation)^31^. For all five of these loci, minor allele frequency (MAF) was higher for those individuals that had lower performance scores (*Χ*^2^ = 9.789, *P* = 0.0017; Fig. 3). We compared 51 individuals born in the 1980s with 57 born between 2005 and 2015 to see whether there had been a change in MAF at these loci over time. All but one locus had diminished MAF over the intervening four or five generations (Table 2). This suggests that there may be purifying selection against these alleles (comparing MAF for all five loci combined, *Χ*^2^ = 2.76, *P* = 0.0483; see Table 2 for details for each locus), but tests involving a longer timeframe would help confirm this. We have no data on the dive performance of the females born in the 1980s, but the range is probably comparable. For our historical genomes, it was possible to genotype eight single-nucleotide polymorphisms (SNPs) among four of these loci (all but *HYOU1*) for three (4 SNPs|) or four (4 SNPs) seals. MAF ranged from 0.125 to 0.167 (average = 0.151) compared with an average of 0.222 among these loci for modern high-performance and 0.307 for low-performance divers. Thus, for our very limited sample, historical MAF is relatively low, as it is for high-performance modern seals, which suggests that it could be due to purifying selection, although drift remains a possibility. More data would be required to make a strong inference about this.

**Table 2:** Genotype data for hypoxia loci in seals showing high and low dive performance

Locus	SNP-ref	Genotype	High P	Low P	Position	MAF-1980s	MAF- ~2010
*HIF1A*	96892683	CC	16	11	Upstream variant	0.44	0.438
		CA	23	21			
		AA	7	14			
	96894173	CC	12	9	Upstream variant	0.45	0.5
		CT	22	20			
		TT	12	17			
*HIF3A*	45676640	AA	38	37	Non-synonymous	0.147	0.052
		AG	7	9			
		GG	1	0			
*SETX*	30169236	CC	42	37	Non-synonymous	0.118	0.052
		CT	4	9			
		TT	0	0			
*HYOU1*	30169060	GG	28	21	Upstream variant	0.275	0.254
		GT	17	21			
		TT	1	4			
*MB*	30169078	CC	37	28	Upstream variant	0.225	0.149
		GC	7	17			
		GG	2	1			

Within each locus, the SNP reference and position relative to the locus is shown. Also shown are changes in MAF comparing females born in the 1980s (*N* = 51) with those born between 2005 and 2015 (*N* = 57).

As a control to test the hypoxia genes results, we used loci of known function (immune system) but for which we expected no correlation with diving performance (upstream SNPs at major histocompatibility complex (MHC) Class 1 *B-alpha*, *CD74*, *DMA* and *DPA1*, and a non-synonymous change in *DQB1*). Five MHC loci were found to have either a variable non-synonymous mutation, or a variable mutation in an upstream region. As expected, no significant correlation was found (*Χ*^2^ = 1.35, *P* = 0.24; Fig. 3). Stochastic distortion of allele frequencies during a bottleneck can increase the frequency of less-fit minor alleles (although none of these showed LOF). These alleles could be retained in the post-bottleneck population if dominance or co-dominance is protective.

## Conclusions

Our results show that the fitness of post-bottleneck northern elephant seals is impacted by stochastic effects and reduced diversity, even though recovery has been rapid, rebounding to a population size comparable to historical maxima. Population bottlenecks are known to distort allele frequency distributions, and distortions have been used to detect bottlenecks^32^. If alleles with stochastically increased frequency are deleterious, they can be maintained in small populations where purifying selection is relatively weak. This can lead to ‘mutational meltdown’ and extinction in asexual species (Müller’s ratchet^33^), but potentially in small populations of sexual species as well^34^. Genomic studies of endangered species have shown the accumulation of LOF loci at levels comparable to what we have found in the post-bottleneck northern elephant seal. For example, in the pygmy hog (*Porcula salvania*), of which there are only a few hundred left, there are substantially more frameshift, stop-gained and missense mutations than in related species^35^.

For the northern elephant seal, we found three categories of post-bottleneck impact. There was a reduction in diversity and an increase in ROH comparing pre- and post-bottleneck genomes, and the loss of diversity was correlated with lower female lifetime reproductive success. Having details on lifetime reproductive success in long-lived animals (as we have here) is rare, but examples of correlations between diversity and fitness proxies are common^36^. The frequency of LOF alleles was also negatively correlated with female lifetime reproductive success. At specific loci associated with reproductive health, LOF allele frequency was correlated with male reproductive success. However, the lack of a genome-wide heterozygosity–fitness correlation and instead a difference associated with MAF at relevant loci for dive performance was especially striking. There was variation within (non-synonymous change) or upstream of five loci associated with hypoxia, not identified as generating LOF. However, those individuals with higher MAF showed lower dive performance, suggesting an impact from post-bottleneck allele frequency distortion. Historical genomes showed lower average MAF than either high- or low-performance divers, and there was an indication of selection against higher MAF over time. We propose that together, these impacts leave the species vulnerable to environmental stresses (such as climate-induced resource bottlenecks)^37^ that an uncompromised population may be able to overcome. In conclusion, our data show that despite rapid recovery and apparent stability, the northern elephant seal has reduced fitness impacting their reproductive output and ability to forage efficiently. Important aspects of impact included the stochastic distortion of allele frequencies and the retention of LOF alleles at critical loci.

## Methods

### Field observation and sampling

Field work was conducted at Año Nuevo State Reserve in California, USA. Details of elephant seal harem observation and tissue sample collection are given in ref. ^3^. Harem observations were for 6–7 hours per day at all harems. A harem was defined as a group of females associated with a single alpha male. The alpha was the highest-ranking male in the dominance hierarchy associated with the harem. Copulations provide a useful measure of reproductive success^38^. Observations at night with a photomultiplier video camera revealed the same rate and pattern of copulations by individuals as during the day^38^. Ronguers were used to take tissue samples from the outer edge of the hind flippers. The samples were stored in 20% dimethylsulfoxide saturated with NaCl^39^. Samples for estimating reproductive success analyses were collected in 1990 and 1991 and those individuals were tagged and tracked over time (between 1981 and 2005), permitting lifetime reproductive success estimates for a subset of females. Details are provided in ref. ^40^. Paternity testing was possible for females attending a given harem that were sampled the following year (a return rate of 40–60% of tagged females).

Data on diving performance were collected between 2004 and 2018. Details on the tags and data collected are provided in ref. ^41^. Tissue samples were again collected from hind flippers and stored in salt/dimethylsulfoxide. All subjects were marked and tagged and recognizable as individuals^42^. Satellite platform transmitter terminals (model ST-6, Telonics) were affixed on the head with marine epoxy, with the antenna angled so that it would be exposed when the seal was at the surface^42^. The transmitter interacted with the ARGOS satellite system to generate locations, which were filtered using standard methods^43^. Data on position, date and time permitted a record of the distance, track and duration of time spent on the foraging trip. A separate tag was affixed to record the depth profiles (a time-temperature-depth-recorder; the MK7 by Wildlife Computers). Data recorded included the age of the seal, the departure and return dates, the mean depth, the maximum depth, the duration and the mass on departure and return. A single foraging trip was recorded for 8 seals, two trips for 68 seals and three trips for 16 seals. We calculated correlations between genomic diversity and a composite measure of diving behaviour. The composite measure was termed dive ‘performance’ and was generated as the average maximum depth (among all trips recorded) times the proportion of dives deeper than the median depth (516 m) times the relative mean duration (compared to the duration of all other dives from the set of 92 individuals). This metric was devised for this study to account for the various aspects of dive endurance recorded.

### Genome sequencing and SNP detection

A total of 260 modern samples were subjected to sequencing. Total genomic DNA was initially extracted using standard phenol/ chloroform extraction and subsequently stored in TE buffer. Short-read libraries for whole-genome sequencing were then constructed as follows. The extracted DNA was sheared into 50–800 bp fragments. Fragments ranging from 150 bp to 500 bp were selected and treated with T4 DNA polymerase (Enzymatics, P7080L) to obtain blunt ends. T-tailed adapters were then ligated to repair these blunt ends. PCR amplification was performed and AMPure XP beads (Agencourt, A63881) were used to purify the PCR products. These short-read libraries were then sequenced on DNBSEQ-T1 sequencers at BGI-Shenzhen, to generate paired-end 100 bp reads. The reference for assembling re-sequencing reads was *Mirounga angustirostris* from DNA Zoo^44,45^ (HI-C, chromosome level from https://www.dnazoo.org/assemblies/Mirounga_angustirostris, total length: 2,366,206,800 bp; scaffold N50: 139,676,048 bp; scaffold N90: 54,920,518 bp, GC content: 41.52%; Supplementary Table 4). The average number of cleaned reads was 1,026.11 million (range: 651.91–7,516.79 million). Percent Q30 was 94.41 on average (range 89.76–97.17; Supplementary Table 5) and average genome coverage was 99.59% (Supplementary Table 6). The average sequencing depth was 33.8X (see Supplementary Fig. 1 for full range). SOAPnuke (v.2.1.5)^46^ was employed for quality control of sequencing reads with parameter ‘-J -l 10 -q 0.1 -n 0.05’ and a config file containing the settings ‘trimBadHead=13,100 trimBadTail=13,100 trim=0,0,0,0 qualSys=2 seqType=0 outQualSys=2’. The clean reads were mapped to the reference genome using BWA^47^ and Realigner in Sentieon^48^, while SNP detection utilized GVCFtyper in Sentieon with default parameters to generate joint-calling of raw SNPs. Sentieon was also used for duplicate read identification and removal (see Supplementary Table 6). The hard filtering of SNPs was performed using VariantFiltration in GATK (v.3.8.1)^49^, with the criterion ‘QD < 2.0 || MQ < 40.0 || FS > 60.0 || ReadPosRankSum < −8.0 || MQRankSum < −12.5 || SOR > 3.0’ as recommended by GATK. Three replicate samples were removed from further analysis.

Historical samples were acquired on loan from the Smithsonian Museum of Natural History, the Harvard Museum of Comparative Zoology and the San Diego Natural History Museum. Samples were bone, tooth or dried dermal tissue. DNA extraction and library construction were done at the Smithsonian Institution’s Center for Conservation Genomics. Work was done in their contained ancient DNA facility using standard precautions to avoid cross-contamination. Extraction followed the protocol in ref. ^50^. Briefly, samples were powdered using a Dremel drill and the calcium extracted from bone and tooth with EDTA (0.5 M, pH 8.0). Samples were then extracted for DNA in extraction buffer (after ref. ^50^ Supplement SD3) overnight at 37 °C with shaking. Solutions were cleaned on Qiagen MinElute columns (28004). DNA quantity was checked on a Qubit 4 fluorometer. DNA libraries were constructed using a KAPA Hyper Prep kit (Roche) following manufacturer protocols. Samples were dual indexed with different pairs of P5 and P7 adapters. Libraries were quantified on Qubit and Tapestation (Agilent) and the results compared.

Historical samples were first sequenced on a MiSeq system (Illumina) to balance quantities on the basis of the number of reads. Reads were mapped against the DNA Zoo NES reference (see above) with Bowtie2 (v.2.3.4.1)^51^ using the ‘–very-sensitive’ setting. After balancing quantities, libraries were pooled for sequencing in two Novaseq 6000 (Illumina) S4 lanes (2 × 100 bp). All historical sample sequencing was done at the Baur Core facility at Harvard University. Total reads per sample are shown in Supplementary Table 1. Sequences were trimmed with BBDuk (sourceforge.net/projects/bbmap/) using these settings: ‘k = 25 mink = 15 edist = 1 ktrim = r rcomp = f t = 8 qtrim=rl trimq = 20 maq = 20’. Quality was on average Q36 and the peak fragment size was in the 50–60 bp range, calculated using FastQC (v.0.11.9)^52^. Using clumpify dedupe in BBtools^53^, duplicate reads were removed. Trimmed and filtered reads were mapped to the NES DNA Zoo reference genome using Bowtie2 with the ‘–very-sensitive’ setting. Mapped reads with a quality score below 10 were removed using SAMtools (v.1.12)^54,55^ ‘view’, and the filtered reads were sorted using SAMtools ‘sort’. Variant sites were called using the ‘mpileup’ and ‘call’ commands in BCFtools (v.1.12)^55^. Using SnpSift^56^ ‘filter’, variant calls with a quality score of 20 and depth below 5 were removed. VCF files were further filtered using BCFtools ‘view’ to include only the 17 identified chromosomal scaffolds.

Mitogenomes were generated for 180 modern adults and all 8 historical samples. The reads with low quality, duplications or adaptors were removed using SOAPnuke (v.2.1.5) (https://github.com/BGI-flexlab/SOAPnuke)^46^, leaving clean reads for the final mitogenome assembly. To normalize the samples, randomly resampling the sequences to 40,000,000 reads from the clean reads of each sample was performed using Seqtk (https://github.com/lh3/seqtk). The mitogenome assembly of the NES was carried out using NOVOPlasty v.3.7, which is a de novo seed-extend-based assembler for organelle genomes^57^. The mitochondrial genomes of the NES (*Mirounga angustirostris*, CM055130.1) and the SES (*Mirounga leonine*, NC_008422.1) were used as seed sequences for the mitochondria assembly and the assembly parameters were set as follows: PE mode, read length = 100, *k*-mer = 39, genome range = 12,000–22,000 and type = mito. The mitogenome tree was based on all historical (8) and all modern adult (180) samples and generated in PAUP (https://paup.phylosolutions.com/) by the neighbour-joining method using the Tamura Nei evolution rate correction and 1,000 bootstrap replicates. It was routed with the outgroup, *Mirounga leonina*.

### Genome analysis

A sliding-window analysis using VCFtools^54^ ‘–window-pi’ measured heterozygous sites every 50,000 bp. The total numbers of heterozygous sites across the genome were measured using the command grep on VCF files and heterozygosity determined by dividing by the total number of sites passing the minimum quality and depth filters for each sample. ROHs were measured for runs greater than 100 kb and 1 Mb long using (for 100 kb) the ‘–bed infile.bed–fam infile.fam–bim infile.bim–homozyg–noweb–allow-extra-chr–homozyg-kb 100–out data’ command in PLINK^58^. Before assessing ROH in modern samples, the VCF file was filtered to remove singletons and minor allele frequencies lower than 0.01.

Demographic history was estimated using the coalescent method implemented in PSMC^59^. Aligned mapped reads (BAM files) for 14 samples (7 males and 7 females chosen randomly within sex and among samples from the 1980s) were converted to consensus sequence in FASTQ format using the samtools/bcftools pipeline. First, the samtools ‘mpileup’ command was used to produce the VCF file from the BAM files, and then through bcftools the consensus sequence was generated with the original consensus calling model. Following that, the vcfutils.pl script was used for the FASTQ conversion: bcftools mpileup -Q 30 -q 30 -f NES.fa sample.bam | bcftools call -c | vcfutils.pl vcf2fq -d 10 -D 100 | gzip > diploid_sample.fq.gz. Mapped reads were filtered for a minimum mapping quality (*q*) of 30 and a minimum base quality (*Q*) of 30. The minimum (*d*) and maximum (*D*) coverages were calculated to allow for vcf2fq, and were set to 10 and 100, respectively (-*d* value to a third of the average depth and -*D* value to twice). The FASTQ file was then converted to the input format for PSMC using the following: fq2psmcfa sample.fq.gz > sample.psmcfa. For the final PSMC command, we used 64 atomic time intervals and 28 (=1 + 25 + 1 + 1) free interval parameters: psmc -p ‘4 + 25*2 + 4 + 6’ -o sample.psmc sample.psmcfa. Finally, the PSMC plot was drawn using the command ‘psmc_plot.pl’, with the per-generation mutation rate ‘-u’ and the generation time in years ‘-g’ set to 2.2 × 10^−09^ and 2.2 × 10^−06^, respectively. One hundred bootstrap replicates were run for each sample and 15/100 samples chosen at random to be included in the plot.

Current effective population size was estimated using the linkage disequilibrium method implemented in SNeP^13^ and GONE^14^. SNeP estimates the historical effective population size on the basis of the relationship between *r*^2^, *N*_e_ and *c* (recombination rate) via linkage disequilibrium estimates using the standard PLINK input file format (.ped and .map files). The squared Pearson’s product-moment correlation coefficient between pairs of loci was used due to the unknown phase of the genotypes. The software uses the physical distance (*δ*) between two loci as a reference and translates it into linkage distance (*d*). We used the default values for minimum distance in bp between SNPs to be analysed ‘-mindist’ and maximum distance in bp between SNPs to be analysed ‘-maxdist’ to 50,000 and 4,000,000, respectively. To infer the recombination rate, we used the ‘-svedf’ flag^58^ as a recombination rate modifier and the default MAF < 0.05, as it has been shown that accounting for MAF results in unbiased *r*^2^ estimates irrespective of sample size^60,61^.

The software GONE^14^ was used to obtain an additional estimate of *N*_e_ based on the linkage disequilibrium method. The VCF file was converted to .ped and .map format using the PLINK software. The parameters for the analysis were set as follows: number of generations 2,000, number of bins 400, no MAF pruning was applied and the maximum value of recombination rate (*c*) was set to 0.05. The number of internal replicates was set to 40 for the programme to provide the geometric mean of the consensus estimate of the historical *N*_e_ out of these replicates.

Correlations between pups weaned per year (a measure of lifetime reproductive success) or diving performance and *F*_ROH_ were done using linear regression. A multiple regression was run using the software at https://stats.blue/Stats_Suite/multiple_linear_ regression_calculator.html. *F*_ROH_ was calculated as the proportion of the genome represented by ROH at the given filter length. LOF loci were identified using SnpEff and the *Mirounga angustirostris* annotation GFF file. There were no good alternatives to using the DNA Zoo NES reference, but it is a high-quality and annotated genome, and the results showed credible patterns. Analyses were split between using only those loci that generated a new stop or caused loss of a start codon and all putative LOF loci discovered (including those of minor effect). For graphing, we coded the homozygote of the LOF allele as ‘1’, the heterozygote as ‘0.5’ and unaffected genotypes as ‘0’, and totalled across all identified loci. SnpEff was also used to identify variation at specific loci of interest, such as those associated with reproduction and hypoxia. We used the following pipeline: java -jar snpEff.jar build -gff3 -v NES.reference based on the annotation file GFF. Following that, we used the command ‘ann’ to annotate the variant file. We used the snpSift toolbox as implemented in snpEff to filter the annotated VCF file on the basis of the effect of the genetic variants, for example, LOF or non-synonymous substitutions (missenses). Although we focused on snpEff, which provides specificity on the nature of the change, we also used the Protein Variation Effect Analyzer (PROVEAN; https://github.com/MonashBioinformaticsPlatform/provean) as an alternative method to test the results. PROVEAN calculates the alignment score of a protein variant by comparing a query sequence with the existing database. To run PROVEAN, we configured it with BLAST v.2.2.31+, CD-HIT v.3.1.2 and the NCBI nr database containing 15,006,980 sequences from 15 August 2011. The tool was run with the default parameters, whereby missense variants with PROVEAN scores greater than or equal to −2.5 were classified as neutral, while those with scores less than −2.5 were considered deleterious on the basis of the default threshold.

To assess kinship, we used the relatedness2 function^54^ implemented in VCFtools. We included 77 pups born in the following year to females, most of whom had been in the focal harems, and determined the number of paternities achieved by each of the 31 males in the sample (including the 4 focal alpha males). Manhattan plot comparisons of high- and low-diving-performance females were generated by writing a trait file and generating the plot in R and TASSEL^62^. There were no clear outliers (data not shown). Data relevant to the LRS and diving analyses are provided in Supplementary Tables 7 and 8.

### Reporting summary

Further information on research design is available in the Nature Portfolio Reporting Summary linked to this article.

### Supplementary information

Reporting Summary

Supplementary Tables 1–8 Supplementary Tables 1–8.

### Source data

Source Data Figs. 1–3 and Extended Data Figs. 1–9 Source data for all figures in one Excel file.

## Figures and Tables

**Extended Data Table 1:**
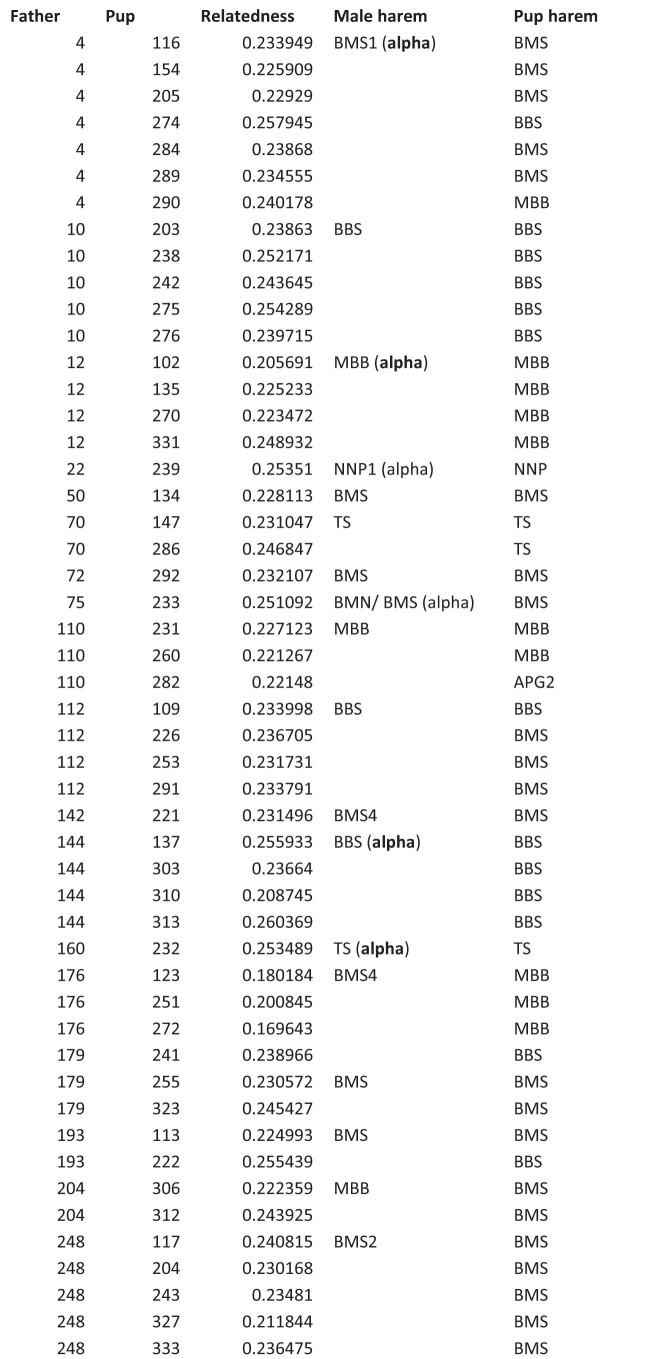
Paternal success of all males

## Data Availability

Sequences are deposited at the National Center for Biotechnology Information (NCBI) under Bioproject PRJNA1060307. Whole-genome data were uploaded as raw reads (Sequence Read Archive), including all historical samples and six high-coverage modern samples (Biosamples SAMN39224291–SAMN39224298; SAMN39305437, SAMN39305439, SAMN39305440, SAMN39305442, SAMN39305447 and SAMN39305448). Variant information for all modern sequences is included as a VCF file (European Variation Archive; PRJNA1060307). All raw nuclear genome data for the modern samples have also been deposited to the China National GeneBank Sequence Archive (CNSA) of the China National GeneBank DataBase (CNGBdb) with accession number CNP0005170. Source data are provided with this paper.
