# Supplementary material for: Genomics of post-bottleneck recovery in the northern elephant seal
Source: Nat Ecol Evol. 2024 Feb 21;8(4):686–94. doi: 10.1038/s41559-024-02337-4 (PMC11009102; doi:10.1038/s41559-024-02337-4)
Supplement: Supplementary file 1 — Reporting Summary [file 41559_2024_2337_MOESM1_ESM.pdf]

nature portfolio

Corresponding author(s): Rus Hoelzel; Shuai Sun; Guangyi Fan; Songhai Li

Last updated by author(s): Jul 4, 2023

Reporting Summary

Nature Portfolio wishes to improve the reproducibility of the work that we publish. This form provides structure for consistency and transparency in reporting. For further information on Nature Portfolio policies, see our Editorial Policies and the Editorial Policy Checklist.

Statistics

For all statistical analyses, confirm that the following items are present in the figure legend, table legend, main text, or Methods section.

n/a	Confirmed	
☐	☒	The exact sample size (*n*) for each experimental group/condition, given as a discrete number and unit of measurement
☐	☒	A statement on whether measurements were taken from distinct samples or whether the same sample was measured repeatedly
☐	☒	The statistical test(s) used AND whether they are one- or two-sided<br>*Only common tests should be described solely by name; describe more complex techniques in the Methods section.*
☒	☐	A description of all covariates tested
☐	☒	A description of any assumptions or corrections, such as tests of normality and adjustment for multiple comparisons
☐	☒	A full description of the statistical parameters including central tendency (e.g. means) or other basic estimates (e.g. regression coefficient) AND variation (e.g. standard deviation) or associated estimates of uncertainty (e.g. confidence intervals)
☒	☐	For null hypothesis testing, the test statistic (e.g. *F*, *t*, *r*) with confidence intervals, effect sizes, degrees of freedom and *P* value noted<br>*Give P values as exact values whenever suitable.*
☒	☐	For Bayesian analysis, information on the choice of priors and Markov chain Monte Carlo settings
☒	☐	For hierarchical and complex designs, identification of the appropriate level for tests and full reporting of outcomes
☒	☐	Estimates of effect sizes (e.g. Cohen's *d*, Pearson's *r*), indicating how they were calculated

Our web collection on statistics for biologists contains articles on many of the points above.

Software and code

Policy information about availability of computer code

Data collection	*Provide a description of all commercial, open source and custom code used to collect the data in this study, specifying the version used OR state that no software was used.*
Data analysis	PSMC; GONe; SNeP; SOAPnuke v2.1.5; BWA; GVCFtyper; GATK v3.8.1; Bowtie2 v2.3.4.1; BBDuk; FastQC v0.11.9; SAMtools v1.12; SnpSift; BCFtools v1.12; PAUP; FASTQ; PLINK; SnpEff; PROVEAN; BLAST; TASSEL

For manuscripts utilizing custom algorithms or software that are central to the research but not yet described in published literature, software must be made available to editors and reviewers. We strongly encourage code deposition in a community repository (e.g. GitHub). See the Nature Portfolio guidelines for submitting code & software for further information.

Data

Policy information about availability of data

All manuscripts must include a data availability statement. This statement should provide the following information, where applicable:

- Accession codes, unique identifiers, or web links for publicly available datasets
- A description of any restrictions on data availability
- For clinical datasets or third party data, please ensure that the statement adheres to our policy

All sequences are deposited on Genbank under project XXXX. There are no restrictions on data availability.

Research involving human participants, their data, or biological material

Policy information about studies with human participants or human data. See also policy information about sex, gender (identity/presentation), and sexual orientation and race, ethnicity and racism.

Reporting on sex and gender	*Use the terms sex (biological attribute) and gender (shaped by social and cultural circumstances) carefully in order to avoid confusing both terms. Indicate if findings apply to only one sex or gender; describe whether sex and gender were considered in study design; whether sex and/or gender was determined based on self-reporting or assigned and methods used. Provide in the source data disaggregated sex and gender data, where this information has been collected, and if consent has been obtained for sharing of individual-level data; provide overall numbers in this Reporting Summary. Please state if this information has not been collected. Report sex- and gender-based analyses where performed, justify reasons for lack of sex- and gender-based analysis.*
Reporting on race, ethnicity, or other socially relevant groupings	*Please specify the socially constructed or socially relevant categorization variable(s) used in your manuscript and explain why they were used. Please note that such variables should not be used as proxies for other socially constructed/relevant variables (for example, race or ethnicity should not be used as a proxy for socioeconomic status). Provide clear definitions of the relevant terms used, how they were provided (by the participants/respondents, the researchers, or third parties), and the method(s) used to classify people into the different categories (e.g. self-report, census or administrative data, social media data, etc.) Please provide details about how you controlled for confounding variables in your analyses.*
Population characteristics	*Describe the covariate-relevant population characteristics of the human research participants (e.g. age, genotypic information, past and current diagnosis and treatment categories). If you filled out the behavioural & social sciences study design questions and have nothing to add here, write "See above."*
Recruitment	*Describe how participants were recruited. Outline any potential self-selection bias or other biases that may be present and how these are likely to impact results.*
Ethics oversight	*Identify the organization(s) that approved the study protocol.*

Note that full information on the approval of the study protocol must also be provided in the manuscript.

Field-specific reporting

Please select the one below that is the best fit for your research. If you are not sure, read the appropriate sections before making your selection.

☐ Life sciences ☐ Behavioural & social sciences ☒ Ecological, evolutionary & environmental sciences

For a reference copy of the document with all sections, see nature.com/documents/nr-reporting-summary-flat.pdf

Ecological, evolutionary & environmental sciences study design

All studies must disclose on these points even when the disclosure is negative.

Study description	All fieldwork was undertaken for earlier studies and published in earlier papers. Samples for DNA analysis were also collected for earlier studies. They were analysed for genome sequences here for the first time. Historical samples were analysed here for the first time.
Research sample	Research samples were collected from Mirounga angustirostris females with known reproductive history, males associated with breeding behaviour data, and females that had been fitted with depth recording devises. Samples were collected from pups from observed harems where the attending males were known to test for paternity. Historical samples were collected before or shortly after the population bottleneck.
Sampling strategy	Sampling strategy targeted animals for which other specific data associated with reproductive success, dive performance or time-frame were available. All modern samples were small biopsy samples collected from the hind flippers with anesthetic and under permit. Historical samples were bone or dried skin.
Data collection	Data collection for this study was collected by observation (reproductive behaviour), telemetry (depth recorders) and DNA sequencing (next generation genome sequencing).
Timing and spatial scale	Field data collection ran from 1981 to 2018 and all subjects were from the breeding colony at Ano Nuevo, CA, USA.
Data exclusions	Only replicate sequences were excluded from the main analysis.
Reproducibility	All repeat sequencing attempts (N=3) were successful.
Randomization	Randomization was not relevant because the object was to compare the genomes of specific individuals (e.g. females with known lifetime reproductive success).

Blinding	Blinding was not relevant because the object was to compare the genomes of specific individuals (e.g. females with known lifetime reproductive success).

Did the study involve field work? ☒ Yes ☐ No

Field work, collection and transport

Field conditions	Modern samples were collected for DNA analysis and transfered quickly to DNA preservative. Samples were refrigerated soon after collection. Historical samples were from museum collections.
Location	All modern samples were collected at Ano Nuevo, CA, USA. Historical samples are also from CA, USA.
Access & import/export	Modern tissue samples were transferred to the UK in the early 1990s. The species is not CITES listed. Historical materials were collected and examined in the US.
Disturbance	Behavioural, interactive and observation studies have been ongoing at this colony since the early 1980s under US government permits. Protocal is designed to minimize disturbance and approved under permit.

Reporting for specific materials, systems and methods

We require information from authors about some types of materials, experimental systems and methods used in many studies. Here, indicate whether each material, system or method listed is relevant to your study. If you are not sure if a list item applies to your research, read the appropriate section before selecting a response.

Materials & experimental systems

n/a	Involved in the study	
☒	☐	Antibodies
☒	☐	Eukaryotic cell lines
☒	☐	Palaeontology and archaeology
☐	☒	Animals and other organisms
☒	☐	Clinical data
☒	☐	Dual use research of concern
☒	☐	Plants

Methods

n/a	Involved in the study	
☒	☐	ChIP-seq
☒	☐	Flow cytometry
☒	☐	MRI-based neuroimaging

Animals and other research organisms

Policy information about studies involving animals; ARRIVE guidelines recommended for reporting animal research, and Sex and Gender in Research

Laboratory animals	No laboratory animals were used.
Wild animals	No animals were killed. Biopsy samples were collected from unrestrained animals, or from animals that were temporarily restrained while telemetry equipment was fitted.
Reporting on sex	The sex of study subjects is identified. Reproductive success is sex-specific, and all telemetry data was from females.
Field-collected samples	Sampling strategy targeted animals for which other specific data were available. All field samples were small biopsy samples collected from the hind flippers with anesthetic and under permit.
Ethics oversight	Fieldwork was conducted under US government research permits.

Note that full information on the approval of the study protocol must also be provided in the manuscript.
